# Parental and Infant Gender Factors in Parent–Infant Interaction: State-Space Dynamic Analysis

**DOI:** 10.3389/fpsyg.2017.01724

**Published:** 2017-10-09

**Authors:** M. Angeles Cerezo, Purificación Sierra-García, Gemma Pons-Salvador, Rosa M. Trenado

**Affiliations:** ^1^Department of Psychology, University of Valencia, Valencia, Spain; ^2^Department of Developmental Psychology, National Distance Education University, Madrid, Spain

**Keywords:** father–infant interaction, mother–infant interaction, parental gender, infant gender, state-space grid (SSG), dynamic systems

## Abstract

This study aimed to investigate the influence of parental gender on their interaction with their infants, considering, as well, the role of the infant’s gender. The State Space Grid (SSG) method, a graphical tool based on the non-linear dynamic system (NDS) approach was used to analyze the interaction, in Free-Play setting, of 52 infants, aged 6 to 10 months, divided into two groups: half of the infants interacted with their fathers and half with their mothers. There were 50% boys in each group. MANOVA results showed no differential parenting of boys and girls. Additionally, mothers and fathers showed no differences in the Diversity of behavioral dyadic states nor in Predictability. However, differences associated with parent’s gender were found in that the paternal dyads were more “active” than the maternal dyads: they were faster in the rates per second of behavioral events and transitions or change of state. In contrast, maternal dyads were more repetitive because, once they visited a certain dyadic state, they tend to be involved in more events. Results showed a significant discriminant function on the parental groups, fathers and mothers. Specifically, the content analyses carried out for the three NDS variables, that previously showed differences between groups, showed particular dyadic behavioral states associated with the rate of Transitions and the Events per Visit ratio. Thus, the transitions involving ‘in–out’ of ‘Child Social Approach neutral – Sensitive Approach neutral’ state and the repetitions of events in the dyadic state ‘Child Play-Sensitive Approach neutral’ distinguished fathers from mothers. The classification of dyads (with fathers and mothers) based on this discriminant function identified 73.10% (19/26) of the father–infant dyads and 88.5% (23/26) of the mother–infant dyads. The study of father-infant interaction using the SSG approach offers interesting possibilities because it characterizes and quantifies the actual moment-to-moment flow of parent–infant interactive dynamics. Our findings showed how observational methods applied to natural contexts offer new facets in father vs. mother interactive behavior with their infants that can inform further developments in this field.

## Introduction

In the last three decades, greater recognition has been given to the role of the father in child development (NIDCH Early Child Care Research Network, 2000; Tamis-LeMonda et al., 2004; Lamb, 2010). Moreover, father involvement predicts the quality of family interactions from the earliest stages of a child’s life (Simonelli et al., 2016). Wider involvement of fathers in the rearing and caring for their infants leads to increased opportunities for early interactions (Pleck, 2010). In this respect, the focus should be on, not only the amount of time the father spends with his infant but also, more importantly, how he uses that time and the quality of the relationship, using objective measures (Yago et al., 2014).

Studying father–infant interactions goes beyond the interest in the specific area itself because the quality of interaction has a strong relationship with the child’s development and attachment (Shaw et al., 2005; Kochanska et al., 2008). In the area of early interaction and attachment, researchers have been traditionally focused on the mother as primary caregiver, to which the use of the Maternal Sensitivity construct has been central (Ainsworth et al., 1974). However, according to Bowlby (1969/1982), the child’s choice of attachment figure depends on who cares for him, or her, and on the composition of the family. Therefore, other people can fulfill the role of primary caregiver. Moreover, the child’s relationships with other figures who share the role of caregiver, along with the primary caregiver, were an area of concern previously noted by Mary Ainsworth herself (Ainsworth et al., 1978).

### Father–Infant Interaction vs. Mother–Infant Interaction

Research findings involving mothers and fathers interacting with babies shows a mixed picture. Some studies, involving children aged from 9 to 24 months, find differences between mothers and fathers. Thus, in contrast with mothers, fathers observed in free-play, were less sensitive and often intrusive, for example, introducing questions or requiring information that may interfere with play (Leaper, 2000; Volling et al., 2002; Lovas, 2005; Kwon et al., 2012; Hallers-Haalboom et al., 2014; Fuertes et al., 2016). Likewise, Feldman and Klein (2003) found that fathers interacting more through contact and physical play, were usually less positive, more unpredictable, and characterized by sudden peaks of emotional intensity. Fathers used more stimulation and exploratory play and less emotional support behaviors (Grossmann et al., 2008).

Other studies, involving a wider range of child ages, from 0 to 36 months, find no differences between fathers and mothers in the quality of interactions with their children (e.g., Goossens and van IJzendoorn, 1990; Braungart-Rieker et al., 2001; Lewis and Lamb, 2003; John et al., 2012; Yago et al., 2014) or in the intensity of their negative affect (Ekas et al., 2011).

These studies involved infants of different ages from very young infants to toddlers (Braungart-Rieker et al., 2001; Yago et al., 2014) and different measures, from event-based schemes to rating scales (Volling et al., 2002; Ekas et al., 2011). Some of these studies compared mother and father with the same child (e.g., Yago et al., 2014) which could be clouding possible differences, as it is plausible that a partner influences the quality of parent–infant interaction. In fact, some research indicates that partners can become similar through the process of marital life (Easterbrooks and Goldberg, 1984; Osnat and Bonnie, 1995; Lundy, 2003). Especially during infancy, parents can rely on each other in searching and implementing successful strategies of interaction with their infant, leading to bidirectional modeling (Braungart-Rieker et al., 1998; Schoppe-Sullivan et al., 2007). Additionally, Barnett et al. (2008) found that perceived high level of marital quality was associated with interdependence of sensitive parenting behaviors in mother–infant and father–infant interactions. This could explain the high correlations between the scores obtained by couples of fathers and mothers in sensitivity and intrusiveness (Braungart-Rieker et al., 1998; Volling et al., 2002; Tamis-LeMonda et al., 2004; Hallers-Haalboom et al., 2014). Finally, most of the studies did not consider infant gender as a factor (Braungart-Rieker et al., 2001; Kwon et al., 2012).

### Gender of Parents and Infants: Early Interaction

Two theoretical frameworks describe some mechanisms regarding differential parenting of boys and girls. The biosocial theory proposes that the parents use gender-differentiated parenting as a means of gender-role socialization (Eagly and Wood, 2002; Wood and Eagly, 2012) and gender schema theories (Bem, 1981; Markus et al., 1982) proposes that parenting would be affected by parents’ gender-role stereotypes.

Considering these two theories, Endendijk et al. (2016), conducted a meta-analysis of 126 observational studies, involving 15,034 families to examine parental differences with their sons and daughters. They used ‘autonomy-supportive strategy’ in parental behavior, that is, child-centered responding and promoting autonomy through support, conceptually similar to the construct of parental sensitivity as formulated within Attachment Theory (Bowlby, 1969/1982; Ainsworth et al., 1978) and controlling strategies, similar to parenting practices described within Coercion Theory (Patterson, 1982; Eddy et al., 2001). Contrary to their expectations, no overall gender-differentiated effect was found in autonomy-supportive strategies, and they found very small effects (*d* = 0.08) of child gender on parents’ use of control after excluding outlying effect sizes by which parents used more controlling strategies with boys than with girls.

Endendijk et al. (2016) in their meta-analytic study included boys and girls from 0 to 18 years. Although biosocial theory does not explicitly consider child age, it is plausible to expect some gender-specific parenting related with developmental level. With older children expressing their demands more clearly, parents would be more effective in adjusting their behavior to their demands. Hallers-Haalboom et al. (2014) reported that mothers responded in a more sensitive and non-intrusive way to their older children (between 2.5 and 3.5 years) compared to younger ones (12 months) without being influenced by infant gender. Likewise, in the area of parental control some meta-analytic evidence supports this, for example, the findings reported by Leaper et al. (1998) by which gender differences in the mother’s directive speech was more evident with older children than with younger ones. In contrast, other studies found that parental control decreases with the child’s age in favor of child self-control (Lytton and Romney, 1991). The combined effect size reported by Endendijk et al. (2016) for the differences in parental controlling: more with boys than with girls was largest in the youngest age group (0–2 years: *d* = 0.16). The findings for that age group were coming mainly from studies involving toddlers, because in the pool of 126 studies, 16.67% (21 studies) included children averaging in age from 1 to 2 years and only one study (Huber, 2012) included children whose average age was under 12 months.

Research suggests that parent–infant interaction can be affected, not only by the gender of children but also the parent’s gender. However, Endendijk et al. (2016) testing only differential controlling of boys and girls in those studies which included fathers and mothers, found no effect of parental gender on the extent of their differential treatment of boys and girls.

In summary, there is no consensus about the extent to which parents treat their sons and daughters differently. Moreover, there are other factors, like the setting of the interaction, which may interact with the gender factor. Thus, in meta-analytic studies it has been found that differences of gender on interaction are often lower in relatively unstructured settings, such as free-play, than in structured tasks such as problem-solving (Leaper et al., 1998; Endendijk et al., 2016).

Therefore, the heterogeneity of measures, age, and settings, across studies in this field can prevent potential gender-differences being detected. However, even the existence of parental differences in the treatment of their children may be reflecting differences in parental practices which may be due to factors other than gender, like birth order (Lovas, 2005; Hallers-Haalboom et al., 2014). Some studies showed that fathers and mothers are more sensitive to the first child than to later ones. These differences were especially pronounced when the second born was the same gender as the firstborn, and fathers were more likely to show differential treatment than mothers (Van IJzendoorn et al., 2000; Furman and Lanthier, 2002; Hallers-Haalboom et al., 2014).

Although studies on dyadic interaction show that the gender of parents and children may affect parental behavior, the direction of those influences is not yet conclusive (Hallers-Haalboom et al., 2014). Nor is it conclusive that the level of parental sensitivity depends, exclusively at least, on the combinations of parent–child gender (Lovas, 2005; Schoppe-Sullivan et al., 2007; Hallers-Haalboom et al., 2014; Endendijk et al., 2016).

### Early Interaction and Measurement

The area of caregiver–infant interaction has been influenced by the Sensitivity construct, referred to as ‘Maternal Sensitivity’ because it has been regarded as one of the most important mediators of attachment patterns (Ainsworth et al., 1978). This construct consists of awareness of the child’s cues and demands, his/her appropriate interpretation, and the ability to respond quickly and accurately (Ainsworth et al., 1978). This central feature of maternal behavior was originally assessed with a global rating scale: Ainsworth’s Maternal Sensitivity Rating Scale (Ainsworth et al., 1974). This strategy has been the most influential and common in this field (for a review of the Global Interaction Scales, see Leclère et al., 2014).

One of the most important features of rating-scale approaches is that they do not capture the temporal dimension of the interaction. Research has highlighted that infants develop an early procedural representation of the world before they develop symbolic forms of representation (Beebe et al., 2010). In addition, the infant’s procedural form of representation is based on his perception of contingency and the predictability of events: infants develop ongoing expectations of sequences of events, within the self, within the ‘other’ and between the two (Tarabulsy et al., 1996; Beebe and Lachmann, 2002; Gergely, 2004). This unfolds during the process of interaction over time. Therefore, to examine these central aspects of parent–infant interaction requires a real time sequential coding approach.

In general, rating-scale and temporal sequential approaches, referred to as macro- and micro-analytic approaches, respectively, have tended to favor different contexts. The macro-analytic one has traditionally used naturalistic situations, such as free-play and the most frequent setting for micro- analytic studies has been face-to-face interaction with the mother on a chair, facing the infant, who is secured in a baby seat. In the latter context, the coding of mother–infant interaction states is done in minor units, for example, units of 1 s. These constraints on the mother–infant interaction and the fragmentation of the analysis have been criticized (Mesman, 2010). In fact, free-play offers greater ecological validity because the mother, or caregiver, has no restrictions on their behavior with their child.

In this context, there is a third way: the observational strategies of sequential coding in real time in a free-play situation (Cerezo et al., 2008). Indeed, the interaction during free-play can be sequentially coded as it unfolds, with mutually exclusive and exhaustive defined categories for infant and mother. Thus, the recorded data can be read as a sort of abbreviated text, reflecting the stream of behavior. The analyses can provide important information, not only about “what” the parent responds to, but also “with what” and “when,” in that stream of social exchange (Cerezo et al., 2012, 2016). Therefore, micro-analytic approaches, that is to say, approaches including the temporal dimension, may be a further step in the understanding of sensitivity and “appropriateness” of parental responses because they look at parental matching/contingent behavior to the child’s behavior, which fosters synchrony and mutual emotional regulation in the interaction (Sroufe, 1995; Feldman, 2007; Woodhouse, 2010).

### Non-linear Dynamic Systems Approach to Interaction

Consideration of the temporal dimension in the measurement of parent–infant interaction allows for the examination of the dynamic process in dyadic interaction. In this context, the non-linear dynamic systems (NDS) framework and its principles that account for properties of dynamic, complex, adaptive, open systems, offer an instrument to examine these processes (Prigogine and Stengers, 1984; Thelen and Smith, 1994, 1998). This approach has led to a paradigm shift in multiple fields (Fogel, 2011), including that of dyadic interaction. Indeed, by accepting the dyad as a dynamic system, then the NDS principles can account for dyadic behavioral patterns that emerge and stabilize through the system’s internal feedback processes (Hollenstein et al., 2004; Hollenstein, 2007; Dishion, 2012). The State Space Grid (SSG) analysis, a graphical tool based on a dynamic system approach (Hollenstein, 2013) allows visualizing the content, temporal and affective flow of the interactions (Sravish et al., 2013) and relevant structures and dimensions of that interaction (Feldman, 2007; Beebe et al., 2010).

Few studies have used NSD and SSG indices in early parent–child interaction. Sravish et al. (2013) have used this paradigm to study the dynamic regulation behaviors between the child and his caregiver through the face-to-face Still-Face paradigm. Cerezo et al. (2012) used the SSG for the study of dyadic flexibility, in the context of interactions. In that study, dyadic flexibility was an index of sensitivity, a precursor of attachment. However, all these studies have focused on mother–infant dyads.

### Antecedents and Purpose of this Study

The present study is part of a research program focusing on detecting precursors of attachment in which the central character has been the mother (Cerezo et al., 2006, 2008, 2012, 2016). The present development of the research program addresses father–infant interaction, compared with mother–infant dyads, using the same observational methodology, the NDS approach and tools of previous studies and, additionally, it considers the factor of the infant’s gender in the interactive process. The general purpose was to progress the understanding of paternal behavior using systematic observation, so the study of precursors of attachment can include fathers when they are caring for their infants, including the potential infant gender effects on that parent–infant interaction.

Given the lack of studies, using this approach for this specific topic, the overall purpose of the current study was exploratory. Specifically, the purpose was twofold: on the one hand, to compare the interactive profile of dyadic temporal organization of fathers with their babies and mothers with theirs and, on the other hand, to examine the effect of the baby’s gender on the interaction.

## Materials and Methods

### Participants

The participants in this study were 52 infants: 26 children interacting with their mothers and 26 children interacting with their fathers. Boys and girls were equally represented (50%) in both groups.

The parents came from the general population who joined a community-based program provided on a universal basis to support parenting during the first 2 years of life. The Program comprised six trimestral visits over a period of a year and a half. As part of the service, parent and infant are videotaped in a free-play situation, with parental consent, to analyze their interaction and provide parents with individual guidance (for a description: Cerezo and Pons-Salvador, 1999; for a summary of evaluation studies: Pons-Salvador et al., 2014). The first time they attend, parents do not receive any information after they have finished the free-play and for more specific and personalized feedback, they need to wait until their second visit when their free-play has been analyzed.

The criteria to select the cases were:

For the parents: to be (a) the biological mother or father, (b) involved with childcare, (c) to be the first visit to the Program; so no previous intervention received and this visit should be before the child was 12 months old. For the infants: (a) absence of congenital anomalies or neurological diseases, all the children’ development was appropriate for their chronological age, assessed by the Developmental Scales of Knobloch et al. (1980) and (b) gender with 50% of girls in each group.

The selection procedure was the following: First, the infants interacting with their fathers were selected. Although the program is offered to both parents, about 15% of fathers attended at least one visit (out of the six Program visits). The initial pool of data comprised 980 families. There were 145 cases of infants interacting with their fathers at least once. From these there were thirty-five who met the criterion of having their first visit to the program on their own and, of those, 29 dyads met the criterion of the child’s age. There were 13 girls in that group; to balance the gender factor thirteen out of the sixteen cases involving interaction with a male infant were randomly selected for the final group. Secondly, to select the group of infants interacting with their mothers, 13 girls were randomly selected from those cases in which mother attended the first visit, so had no prior intervention and then 13 boys to comprise a similar group to the one of infants with their fathers.

In both groups, mothers’ and fathers’ interaction was assessed when their children were, on average, 36.47 weeks of age (*SD* = 2.85), ranging from 26 to 44 weeks. No significant differences in infants’ age (*t* = 3.37, *df* = 48, *p* = 0.71) with mothers: *M*_age_ = 36.62, *SD*_age_ = 2.46 and with fathers: *M*_age_ = 36.32, *SD*_age_ = 3.22. The second half of the first year shows important advances for the child’s social and emotional development. From the neuro-relational approach, regarding adjustment and interaction, Lillas and Turnbull (2009) point to the age range 6 to 10 months as being the one where children display bi-directional intentional communication (child–adult) interaction.

The mean age of the 26 mothers in the study was 28.35 years (*SD* = 6.09), ranging from 17 to 41 years. As for the 26 fathers, the mean age was 32.48 years (*SD* = 6.76), with a range between 21 and 48 years. The two groups had a similar average number of children: 1.58 (*SD* = 1.03) in the group of mothers and in the group of fathers, 1.73 (*SD* = 1.18). The birth order for infants was similar in both groups. Thus, the majority of the infants were first or second born: 84.61% and 88.46%, in father and mother groups, respectively, 11.53 and 7.69% were third or fourth born, and only 3.84% in both groups were children in fifth or sixth position. The comparison between mother and father groups in the number of dyads with first child vs. second child vs. third child plus, showed no significant differences. In the father–infant group, all the fathers, except one, came from two-parent families and there were three single-mothers in the mother–infant group.

The participants were all resident in Ireland. In the fathers group 84.61% were Irish or from other European countries, like Poland, and the rest, 15.39%, were from the United States, the Philippines, Libya, and Turkey. With regard to the mothers, 76.92% were Irish or from other European countries. There were 7.69% mothers from South-American countries, 11.53% from African countries and 3.8% from India.

Regarding educational levels, 69.23% mothers and 42.3% fathers had only Secondary School education (Intermediate/Junior Cert level: 26.92% of mothers and 7.69% of fathers; Leaving Certificate level 42.31% mothers and 34.61% fathers). Studies at third level: 23.10% of mothers and 34.61% of fathers, finally 7.69% of mothers and 11.53% of fathers reported post-graduate studies. One father reported only having Primary school studies (3.84%) and two fathers did not provide this information (7.69%).

Regarding occupations, there were 11.53% mothers working full time at home. In the two groups, 53.84% of mothers and 46.15% of fathers reported being unemployed, 11.53% mothers and 7.69% of fathers worked in unskilled occupations, while 15.38% of mothers and 30.76% fathers were in semi-skilled jobs, and, finally, 11.53% in each group were in qualified occupations.

### Procedures

In the context of the visit to the Program, the professional left the parent with the infant in a room for the free-play. This session took place in a room with a table and a chair and there were some toys appropriate for the child’s age. The parent was told to play with his/her child the way she/he normally did and if she/he wanted, she/he could use the toys. The systematic observation was carried out on 4–5 min free-play session. An average of 5 min play is sufficient, according to studies in this area by Kemppinen et al. (2005). On the table there was a matt and in all cases parents played with their child on the table or on their lap. The session was videotaped for coding as part of the routine of the Program. Consequently, the protocol for the staff was that the parent and child play between 4 and 5 min, with flexibility. The final duration did not have to do with any parental/infant characteristic. Sometimes, the play was closer to 4 min and sometimes a bit longer, depending on the circumstances (i.e., staff may have been momentarily busy when the timer rang went off).

The parents were informed consent to be videotaped. Additionally, they gave written consent for the anonymous use of their data for research purposes. This study was approved by the Research Ethics Committee of the University of Valencia.

### Measures

#### Parent–Infant Interaction and Early Mother–Child Interaction Coding System-Revised (Códigos para la Interacción Temprana Materno-Infantil: CITMI-R (Trenado and Cerezo, 2007, Unpublished)

**FIGURE 1:**
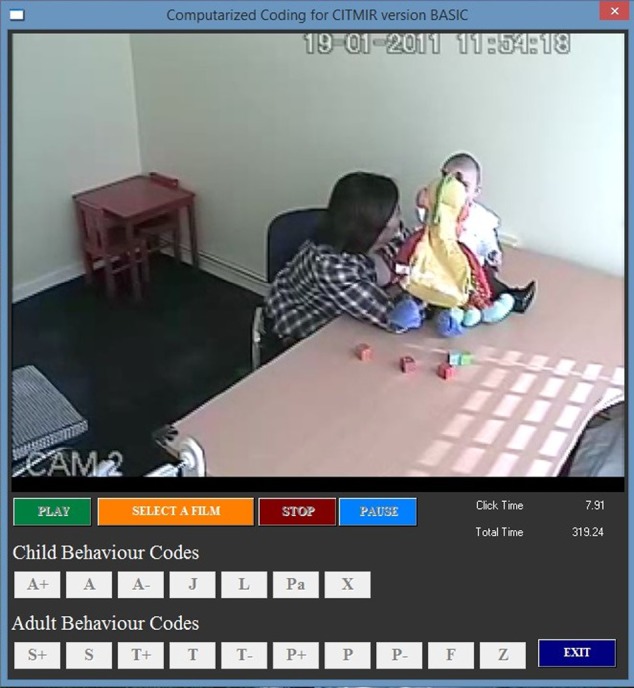
Illustration of CITMI-R computerized coding system applied to a Free-Play recording.

The structure and coding rules of CITMI-R are based on SOC III (Cerezo, 2000), being a parallel version for young children. The CITMI-R categories, having been defined in a mutually and exhaustive way, put the stream of mother–child interaction into observational data that can be analyzed. The computerized coding, specially devised for CITMI-R coding, allows the observer to code in real time without interruption during the period of observation (**Figure 1**).

CITMI has shown good standards of psychometric properties in content validity and criterion validity with dyads from Spain, Brazil, and Ireland (Alvarenga and Cerezo, 2013; Trenado et al., 2014).

#### The CITMI-R Observational Categories

It includes four categories for the parent’s behavior, three interactive and two non-interactive (**Table 1**). For the child there are four categories, one interactive and three non-interactive. All interactive behaviors, according to affect, can either be positive, neutral, or negative, except for “Sensitive” parental behavior, which, by definition and nature, can only be either neutral or positive affect. Therefore, there were 54 possible dyadic states, “parent–infant variables,” used for SSG: nine maternal/paternal codes ×6 infant’s codes. **Table 1** shows a descriptive summary of the CITMI-R.

**Table 1:** Summary of the categories in the *Early Mother–Child Interaction Coding System-Revised* (CITMI-R).

Child categories
**Interactive**
**Social approach (A):** Social approach, verbal, or non-verbal, to the parent as a response to her/him or as a child’s initiative. It has three affect or valences: positive, neutral, and negative.
**Non-Interactive**
**Solitary play (J):** The child is involved in his/her own game with or without a toy; she/he is clearly demonstrating interest in the exploration (his own hands, clothes, objects, etc.).
**Solitary crying and/or whining (L):** The child express general discomfort usually related to being tired, sleepy or hungry.
**Passive/disinterested/apathetic behavior (Pa):** The child shows a bored or non-attentive facial expression. If the child ever catches hold of something there is not looking at it or exploration.
**Parental categories**
**Interactive**
**Sensitive (S):** Social approach, verbal or non-verbal, that meets the demands of the situation and is appropriate for the age, abilities and interests of children. This approach DOES NOT interrupt child’s ongoing activity, or intrude in child’s space. It includes proposals of toys or games to the child in a way that the child has a choice to accept it or not. It has two affect or valences: positive and neutral.
**Intrusive (T):** Social approach, verbal or non-verbal, that interrupts on-going activities of the child and/or invades his space and it is not meeting child’s needs. It includes proposals of toys or games to the child in a way that the child has no choice or they are above child’s skills or reach, like putting a toy in his hand, or too far away. It has three affect or valences: positive, neutral, and negative.
**Protective (P):** Social approach, verbal or non-verbal that interrupts child’s ongoing activity, or intrudes in child’s space with the aim of protection or help (wiping child’s nose, changing child’s position, etc.). It has three affect or valences: positive, neutral and negative.
**Non-Interactive**
**Indifferent/non-response (F):** Lack of interaction with the child showing lack of attentiveness and lack of facial expression, or the parent looks away not responding to child’s approach.

#### NDS and State Space Grid Measures

**FIGURE 2:**
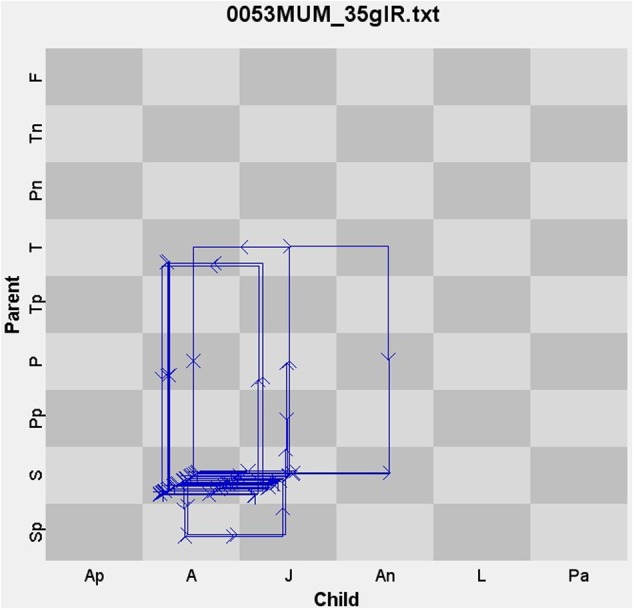
Example of an individual trajectory, a dyadic interaction, in the state-space grid.

The unit was the dyad. The codes for the infant (*x*-axis) and the parent (*y*-axis) were represented on a quasi-ordinal scale from the most positive to the most negative (**Figure 2**). The state-space grid for this study consisted of 54 (cells) potentially possible dyadic states: any combination of parent–child behavior. Each dyad “danced” around the state-space grid during the Free-Play session, which was considered as an individual trajectory. Each trajectory begins in one state (cell) and, as time progresses, tends to visit other cells on the grid (**Figure 2**).

The variables derived from the SSG data were:

(a) Different states that the dyad visit in the total state-space grid. A greater number of states (cells) visited means a greater range of content. The variable operationalized was the number of different cells occupied: ‘Diversity’ the value of which could go from one to 54 possible states.
(b) “Dispersion” in the state-space grid: a relative value, the sum of the squared proportional duration across all the cells. The values range from 0 (no dispersion at all - all behavior in one cell, therefore highest predictability) to 1 (maximum dispersion, less predictability). “Dispersion” strongly correlates with duration entropy (Hollenstein, 2013). The value is created by the formula: $1-\left(\left(n\sum {\left(d_{i}/D\right)}^{2}\right)-1\right)/\left(n-1\right)$. Where *D* is the total duration, *d*_i_ is the duration in the cell *i*, and *n* the total number of cells.
(c) Dyadic event corresponded to a single node in a particular cell. Dyadic events are the smallest unit of states displayed on the grid and can have different durations. It was operationalized as the ‘Frequency of Events’ registered.
(d) Changes from one state to another, transitions or movements between cells on the grid done by the dyad. This is a content independent and dynamic measure of variability because transitions can take place between any numbers of cells. A cell visit beginning upon a trajectory’s entry into the cell and ending upon its exit. There can be one or more consecutive events occurring within a single visit. The variable operationalized was ‘Transitions,’ number of visits, minus 1, because the first event in the first cell is counted as a visit.
       ‘Events’ and ‘Transitions’ are frequency-based measures. Therefore, they are time dependent, the longer the observation the more opportunities. Consequently, it is recommended that the variables of frequency of Events and frequency of Transitions be divided by the total duration of the trajectory because the duration of each trajectory – time observed in each dyad - can be slightly different, Thus the variables were converted into rates per second: ‘Transitions rate’ and ‘Events rate.’
(e) Events per Visits Ratio. When there are no repeating events, the ‘Events per Visits’ ratio is 1, the higher the ratio, the more repetitive events take place.

#### Reliability for Interactive Measures

The quality of the data coded from the free-play was validated by having a second independent coder, who was unaware of the study’s purpose, coding one third of the total number of free-play episodes. These 16 dyads were randomly selected, half from the father–infant group and half from the mother–infant group. For the purpose of the reliability analyses, the second coding had the same length as the first, to avoid differences between the outputs in the length of the coded observation. The average length of the observation across the 16 dyads was 5.2 min (*SD* = 1.3).

Specifically, for the reliability analysis of the measurements obtained by CITMI-R, three approaches were used. Firstly, Alignment Kappa (Bakeman and Quera, 2011) was computed to calculate the agreement between coders. This method identifies commission-omission errors and is based on an algorithm that determines the optimal global alignment between two single code event sequences. The mean Kappa statistic for the 16 episodes analyzed, and for all categories, was 0.68 (*SD* = 0.06). The values from 0.61 to 0.80 are considered good and observer accuracies of 90% or better result in alignment kappa of 0.60 or better (Quera et al., 2007). Secondly, the SSG was used and the main variables derived were considered to test the reliability in terms of agreement between the two observers on the NDS variables under study. The Pearson correlations were computed for “Diversity,” *r*_xx_ = 0.74, mean scores for coders 1 and 2: 8.19 (*SD* = 2.85), and 8.56 (*SD* = 2.63); “Duration per cell,” *r*_xx_ = 0.84, mean scores for coders 1 and 2: 44.37 (*SD* = 23.05), and 45.21 (*SD* = 25.33); “Number of Events,” *r*_xx_ = 0.91, mean scores for coders 1 and 2: 126.94 (*SD* = 29.58), and 112.50 (*SD* = 25.32) and, finally for “Number of Visits,” *r*_xx_ = 0.71, mean scores for coders 1 and 2: 50.06 (*SD* = 16.02), and 42.13 (*SD* = 15.42). All the correlation values were shown to be statistically significant (*p* < 0.002). Thirdly, given that Intraclass Correlation Coefficient (ICC) is also recommended (Fleiss and Cohen, 1973; Shoukri, 2004), this was also computed for the same SSG variables. The following values were obtained: “Diversity,” ICC = 0.85; “Duration per cell,” ICC = 0.91; “Number of Events,” ICC = 0.94; “Number of Visits,” ICC = 0.83. All values were shown to be statistically significant (*p* < 0.001) and can be interpreted as excellent, as all were above 0.80.

### Data Analyses

The SSG analysis (Lewis et al., 1999; Hollenstein, 2013) allows visualizing the content, temporal, and affective flow of the interactions (Sravish et al., 2013) and relevant structures and dimensions of the interaction (Peck, 2003; Feldman, 2007; Beebe et al., 2010).

The parental and infant gender factors on parent–infant interaction were examined by conducting a two-way MANOVA (Multivariate analysis of variance) for the data obtained with the SSG regarding ‘Diversity,’ ‘Dispersion,’ ‘Event rate,’ ‘Transition rate,’ and ‘Events per Visit’ ratio. The assumption of homogeneity was tested by computing variance Levene’s Test.

To study the magnitude of the relationships between the variables analyzed significance level value of 0.05 was considered, and the effect size statistic (η^2^) was computed. The statistical package SPSS v.21 for Windows was used for the analyses.

As part of the analysis plan, if the SSG variables showed differences between groups, it was planned to analyze the 54 possible potential dyadic states for those particular variables, to predict father–infant dyads vs. mother–infant dyads. For this purpose a Linear Discriminant Analysis was chosen with a stepwise variable selection method, applying the Wilks Lambda method, and the verification criteria associated with the *F* values by default, in SPSS program.

## Results

After a preliminary analyses section, the results section regarding parental and infant gender on parent infant interaction will address the two goals of the study. Firstly, the study of the NDS and State-Space measures considering parental and infant gender and secondly, to examine the NDS and State-Space measures considering the behavioral dyadic states to predict parental gender membership of the dyads.

### Preliminary Analyses

The duration of the Free-play ranged from 240.05 to 348.60 s. As a preliminary step, the observation time was analyzed in relation to parental and infant gender using ANOVA. The dependent variable was the duration of the free-play.

The results showed that there were statistically significant differences in the observation time between the groups of dyads. The free-play from the father–infant dyads was shorter (*M* = 267.58, *SD* = 13.69) than the one involving mothers and infants (*M* = 321.07, *SD* = 13.69), (*F*(1,51) = 7.83, *p* = 0.008, η^2^ = 0.14). No differences were found for the infant’s gender (*F*(1,51) = 0.70, *p* = 0.40, η^2^ = 0.015), or parental gender by infant gender (*F*(3,153) = 0.02, *p* = 0.90, η^2^ = 0.00). Consequently, for subsequent analyses the number of events and transitions, based on the number of visits, were divided into the observation time in seconds for each dyad, using the rate per second.

### Parental and Infant Gender on the Parent–Infant Interaction: NDS and State-Space Variables

**FIGURE 3:**
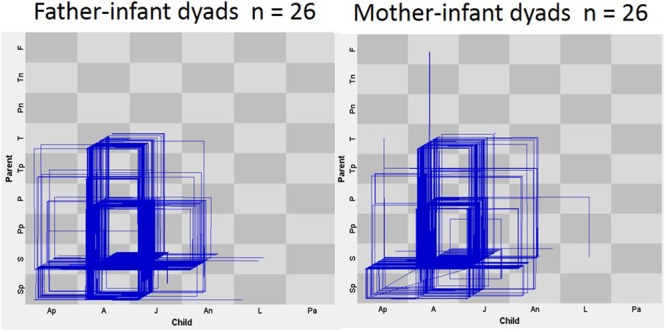
State-space grids (SSG) for each group: father–infant dyads (left) and mother–infant dyads (right). The twenty-six dyads, trajectories, are represented in each SSG.

The two-way MANOVA to test parental gender and infant gender on the parent–infant interaction measures showed no interaction effect between the two factors: parental and infant gender on the combined dependent variables. There was a multivariate effect for parental gender on the parent–infant interaction measures (*F*(5,44) = 6.52, *p* = 0.00, Wilk’s λ = 0.563, η^2^ = 0.43) (**Figure 3**).

**FIGURE 4:**
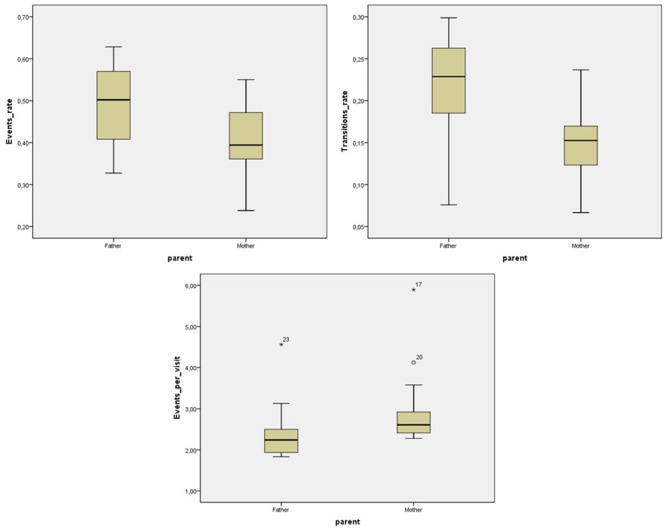
State-space grids measures that showed statistically significant differences between father–infant and mother–infant groups. From the top left: Events rate, top right: Transitions rate, and bottom: Events per Visit ratio.

Specifically, significant differences were found in the Event rate (*F*(1,39) = 12.34, *p* = 0.001, η^2^ = 0.20) and in the Transition rate (*F*(1,39) = 23.23, *p* < 0.001, η^2^ = 0.33), in both of those the father–infant dyads showed a higher rate per second than the mother–infant dyads. Moreover, the two groups showed significant differences in the Events per Visits ratio (*F*(1,39) = 7.09, *p* = 0.010, η^2^ = 0.13). The mother–infant dyads showed a higher value than the father–infant dyads (**Table 2**). Taken together the results indicated that dyads with fathers changed more frequently from state-to-state and engaged in more events per second, while mothers were more repetitive than fathers when interacting with their infants (**Figure 4**).

**Table 2:** Descriptive statistics and between-group comparisons.

NDS variables and SSG measures	Father–infant dyads			Mother–infant dyads			Father–infants vs. Mother–infants groups		
	Total	Boy	Girl	Total	Boy	Girl			
			
	*M (SD)*	*M (SD)*	*M (SD)*	*M (SD)*	*M (SD)*	*M (SD)*	*F*	*p*	*η^2^*
Diversity	9.38 (3.29)	9.38 (2.98)	9.38 (3.71)	8.19 (2.65)	7.31 (2.69)	9.07 (2.39)	2.07	0.15	0.04
Dispersion	0.68 (0.12)	0.68 (0.13)	0.68 (0.11)	0.63 (0.11)	0.59 (0.13)	0.66 (0.09)	2.89	0.10	0.06
Events rate (seconds)	0.49 (0.09)	0.51 (0.09)	0.47 (0.08)	0.40 (0.08)	0.41 (0.07)	0.40 (0.09)	12.34	0.00^∗∗^	0.20
Transitions rate (seconds)	0.22 (0.05)	0.22 (0.06)	0.21 (0.04)	0.15 (0.04)	0.15 (0.03)	0.15 (0.05)	23.23	0.00^∗∗^	0.33
Events per visits ratio	2.34 (0.56)	2.44 (0.73)	2.25 (0.35)	2.84 (0.76)	2.74 (0.40)	2.94 (1.0)	7.09	0.01^∗^	0.13

*^∗^*p* < 0.05, ^∗∗^*p* < 0.001.*

Regarding exploratory examination about the possible differences in interaction with infant boys and girls, no significant differences were found in relation to infant gender (*F*(5,44) = 0.93, *p* = 0.47, η^2^ = 0.09). Therefore, in terms of the measures examined in this study, parent–infant interaction was similar with boys and girls.

In summary, both mother and father dyads showed similar diversity in their interaction, in terms of the number of different dyadic states they went through, and similar levels of dispersion. Additionally, paternal dyads were more ‘active’ than maternal dyads: they were faster in the rate of Events and in the rate of Transitions (per second). In contrast, maternal dyads were more repetitive than paternal ones because they engaged in more events once they visited a particular dyadic state. There were no differences between girls and boys.

### Profile of the Dyadic States Considering the Parental and the Infant Gender

Discriminant analyses were used to determine the linear combination of SSG variables that best classified the 52 dyads into each of the two groups: with fathers and with mothers. We established regarding previous probabilities that all groups were equal. Therefore, based on a discriminant analysis of ‘Events rate,’ ‘Transitions rate,’ and ‘Events per Visit’ ratio, functions were derived for the total grid. These variables were selected because they showed significant differences, and the purpose was to examine, in terms of content, what state or states (dyadic behaviors) could potentially distinguish the two type of dyads. The Eigenvalues, relative variance, canonical correlations and significance tests are shown in **Table 3**.

**Table 3:** Linear Discriminant Analyses results, variables included in the stepwise discriminant analysis and summary of classification results.

Function	Eigenvalue	% variance	Canonical correlations	Wilks’ lambda	Chi-square	*df*	*p*
1	0.46	100	0.56	0.68	18.44	8	0.000

**Steps**	**Variables**		**Standardized coefficients**	**Wilks’ lambda**	***F***	***df***	***p***

1	Transitions rate AS		1.206	0.75	16.49	(1, 50)	0.000
2	Events per visits ratio JS		-0.637	0.68	11.19	(2, 49)	0.000

			**Predicted group membership**		**Total**		
			Father	Mother			
Original group membership		Father	19 (73.1%)	7 (26.9%)	26		
		Mother	3 (11.5%)	23 (88.5%)	26		
		80.8% of original grouped cases correctly classified.			

As **Table 3** shows, overall the Wilks Lambda value is moderately high (0.68), and the Lambda transformed value showed a statistically significant level [χ^2^(2,*N* = 52) = 18.44, *p* = 0.000], which supported the rejection of the null hypothesis. Therefore, the means of the father–infant dyads and mother–infant dyads on the discriminant function –the centroids, were significantly different. Likewise, the variance in the dependent variable accounted for by this model was 46%.

The stepwise discriminant analysis included two variables out of the three analyzed in the following order: ‘Transitions rate’ for the dyadic behavioral state ‘Child Social Approach, neutral-Sensitive Approach neutral’ (TR-AS) and ‘Events per Visit’ ratio for ‘Child Play-Sensitive Approach neutral’ (ER-JS).

**FIGURE 5:**
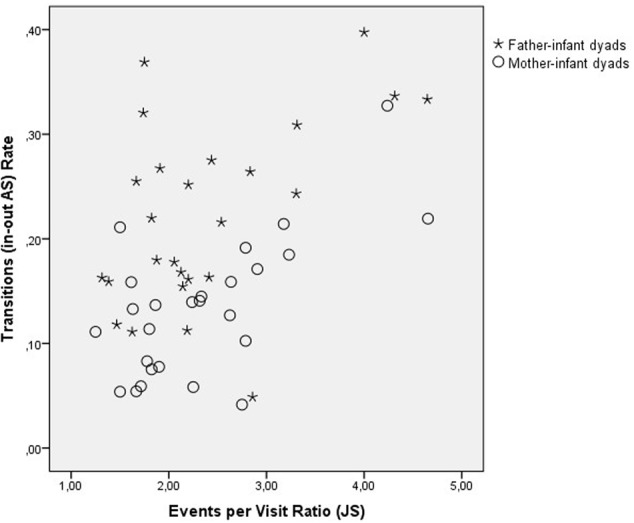
Scatter plot using the two discriminant dimensions: ‘Transitions rate’ for the dyadic behavioral state ‘Child Social Approach, neutral-Sensitive Approach neutral” and ‘Events per Visit’ ratio for ‘Child Play-Sensitive Approach neutral.’

The standardized discriminant function coefficients indicated the relative importance of the independent variables in predicting group membership. This function was marked by a positive coefficient for TR-AS and negative weight for ER-JS. Thus, the lower the TR-AS and the higher the ER-JS the less likely it was that the dyad was from the father–infant group and more likely to belong to the mother–infant group (**Figure 5**).

#### Classification Results Based on the Discriminant Function as Predictor

The classification of dyads (with fathers and mothers) based on this discriminant function for the two groups of parents, showed that 80.8% (42/52) of all the cases were correctly classified as compared with a chance classification of 50%. The function identified 73.10% (19/26) of the father–infant dyads and 88.5% (23/26) of the mother–infant dyads.

In summary, the content analyses carried out for the three NDS variables that previously showed differences between dyads involving fathers and mothers, indicated that there was no particular dyadic behaviors associated with the differences in terms of Events rate per second. However, there were particular states associated with the Transitions rate and the Events per Visit: The transitions involved the ‘in–out’ ‘A-S’ state (‘Child Social Approach, neutral-Sensitive Approach neutral’) and the repetitions of events involving ‘J-S’ (‘Child Play-Sensitive approach neutral’). The combination of both in the discriminant function distinguished dyads with mothers from dyads with fathers.

## Discussion

Our results showed no differences between boys and girls in the parent–infant interaction, regardless of the parent’s gender. This lack of differential parental interaction of boys and girls found in both groups of dyads, with fathers and with mothers, was partially congruent with the meta-analytic study results reported by Endendijk et al., 2016. They found no overall child gender-differentiated effect in parental autonomy-supportive strategies, conceptually similar to the Sensitivity construct. However, in those findings only the factor of the child’s gender was considered.

Subsequently, those authors only selected the twenty-five studies that included mothers and fathers to test the parent’s gender effect, and focused on the controlling strategies that had shown significant differences for the child’s gender. They reported no parent gender effect in the extent of their differential treatment in controlling strategies with boys and girls. Regarding this parental control, some findings indicated that gender differences, in the use of parental control strategies are less relevant when the children are younger (Lytton and Romney, 1991; Leaper et al., 1998; Alink et al., 2006; Else-Quest et al., 2006). However, in Endendijk et al. (2016) meta-analysis the size effect was more relevant in the group aged 0–2. The infants in the present study ranged from 6 to 10 months. One possible reason to explain the discrepancy with our results could be that in the twenty-one studies that comprised their 0–2 age group, only (Huber’s (2012) study included children averaged under 12 months, the rest of them included toddlers, a developmental period for which parental controlling strategies are more relevant.

Other factors that need to be considered in the interpretation of our findings are the setting, i.e., free-play, and socio-economic status (SES). The free-play setting is relatively unstructured and in these settings gender differences in interaction are often lower than in structured tasks such as problem-solving (Endendijk et al., 2016). Additionally, our participants, in sociodemographic terms, were characterized by low-SES. According to biosocial theory (Eagly and Wood, 2002; Wood and Eagly, 2012) and gender schema theories (Bem, 1981; Markus et al., 1982), lower status would tend toward a more traditional division of roles which would result in a greater differentiation of gender roles that transmit into their parental practices. However, our findings did not support this. It could be that the division of gender roles has softened in the Western world (Cabrera et al., 2000; Lamb, 2010) and, as a result, has produced more egalitarian societies (Inglehart et al., 2003). In this regard, the date of publications has shown a significant association with their reported findings about differential parenting for boys and girls (Endendijk et al., 2016). The lack of child gender differences in interactions found in the present study may be due to the young age, under 10 months, of the infants and the free-play setting that is related with lower child gender differences in interaction and this was shown to be the case for both fathers and mothers.

The lack of consensus about the extent to which parents treat their sons and daughters differently can be partially explained by the wide range of child ages included in the studies, the variety of measures and observational strategies and settings. Future studies controlling these relevant factors will shed light on this particular issue.

In relation to mothers vs. fathers, the findings of the present study showed that infants interacting with their fathers and infants interacting with their mothers were involved in a similar number of different dyadic states and their interaction showed similar medium–high levels of predictability. The latter finding runs contrary to the findings of Feldman and Klein, (2003) who reported that fathers were more unpredictable than mothers were. However, the fact that the study was conducted with toddlers, in a compliance situation, could explain this discrepancy.

The differences associated with parent’s gender showed that the dyads involving fathers, compared with their counterparts involving mothers, were having more back-and-forth per unit of time, i.e., Events rate and the discriminant analyses showed no particular type of event, in terms of behavioral content, distinguishing the two groups of dyads. The dyads with fathers were more active, as well, in changing from one type of dyadic state to another, per unit of time, i.e., rate of Transitions; analyses indicated that the behavioral dyadic state involved was ‘A-S’ (‘Child Social Approach, neutral-Parent Sensitive Approach, neutral’). These findings seem to be in line with those that reported that fathers use more stimulation in terms of activating interaction (De Wolff and van IJzendoorn, 1997; Grossmann et al., 2008).

In contrast, dyads with mothers showed more repetition of the same dyadic event, once they moved into a particular state. Further analyses indicated that the visits to the behavioral state ‘J-S’ (‘Child Play-Parent Sensitive Approach neutral’) was the one where mothers were more likely to have more frequency of J-S exchanges, i.e., events, before they move to another state. Taken together the two factors, the Transitions rate of A-S and the Events per Visit ratio of JS, comprised the discriminant function that correctly classified 80.1% of the 52 dyads.

The field of father studies is receiving increasing attention in latter decades. However, the specific area of studies using observational measures of paternal interactive behavior with infants is still very limited. The focus is on comparing fathers with mothers to examine the influence of parental gender on their dyadic interaction and to considering, as well, the potential role of the infant’s gender. However, progressing knowledge of paternal interactive features and sensitivity that may link with child attachment development is of particular interest for child development studies.

However, although the general sense is that gender of parents and children may affect parental behavior, the direction of those influences is not yet conclusive. Moreover, it is not conclusive, either, that the level of parental sensitivity depends, at least exclusively, on the combinations of parent–child gender (Lovas, 2005; Schoppe-Sullivan et al., 2007; Hallers-Haalboom et al., 2014; Endendijk et al., 2016). In this sense, future studies should specifically consider other factors like birth order, controlling interdependence effects between the parents.

The study of father–infant interaction using the SSG approach offers interesting possibilities because it characterizes and quantifies the actual moment-to-moment flow of infant–parent interactive dynamics. Our findings showed new facets in father vs. mother interactive behavior with their infants that can inform further developments in this field.

### Limitations and Strengths

This study presents some limitations. Firstly, the groups who came from nonclinical populations included a proportion of individuals from diverse cultures; about 20% were from India, Turkey, Libya, or African countries. As caregiving behavior may be influenced by cultural factors in both fathers and mothers, some caution needs to be taken in generalizing the reported findings of the present study. Secondly, about half of the mothers and the fathers in this study reported being unemployed. No assessment of factors like depression or depressive mood, due to economic stress, was done, and this might have an effect on their interaction with their infants.

This study also presents some strength. It involved fathers and mothers, each interacting with their own infant, this controls for possible interdependence effects between the parents. The age of the infants was between 6 and 10 months, which reduces the possible interference of using a wide age range. Moreover, this age is very relevant to study interactive patterns that can be antecedents to the quality of child attachment. The number of girls and boys was the same in both groups of dyads with fathers and mothers. The participants came from a general population who joined a community-based program provided on a universal basis. Finally, the analytical approach using SSG methodology with dyadic variables allows for the characterization of different temporal features of father interaction with quantitative measures that can shed light on the paternal sensitivity construct.

## Author Contributions

MC, contributed to the conception of the work, acquisition of data, state-space grid analysis, interpretation of data and revising the work for intellectual content. PS-G, contributed to the design of the work, interpretation of data and revision. GP-S, contributed to the preparation of files and analyses testing the parental and infant gender factors on parent-interaction. RT, contributed to the coding data collection and analysis of the reliability of the observational measures. All authors contributed to drafting the work and gave their approval to the final version to be published. They also agreed to be accountable for all aspects of the work in ensuring that questions related to the accuracy or integrity of any part of the work was appropriately investigated and resolved.

## Conflict of Interest Statement

The authors declare that the research was conducted in the absence of any commercial or financial relationships that could be construed as a potential conflict of interest.
